# Large Language Models for Supporting Clear Writing and Detecting Spin in Randomized Controlled Trials in Oncology: Comparative Analysis of GPT Models and Prompts

**DOI:** 10.2196/78221

**Published:** 2026-01-21

**Authors:** Carole Koechli, Fabio Dennstädt, Christina Schröder, Daniel M Aebersold, Robert Förster, Daniel R Zwahlen, Paul Windisch

**Affiliations:** 1 Department of Radiation Oncology, Kantonsspital Winterthur, Brauerstrasse 15, Winterthur, Switzerland, 41 52 266 26 53; 2 Department of Radiation Oncology, Inselspital, Bern University Hospital, University of Bern, Bern, Switzerland

**Keywords:** spin, randomized controlled trials, large language models, data mining, natural language processing

## Abstract

**Background:**

Randomized controlled trials (RCTs) are the gold standard for evaluating interventions in oncology, but reporting can be subject to “spin”—presenting results in ways that mislead readers about true efficacy.

**Objective:**

This study aimed to investigate whether large language models (LLMs) could provide a standardized approach to detect spin, particularly in the conclusions, where it most commonly occurs.

**Methods:**

We randomly sampled 250 two-arm, single–primary end point oncology RCTs from 7 major medical journals published between 2005 and 2023. Two authors independently annotated trials as positive or negative based on whether they met their primary end point. Three commercial LLMs (GPT-3.5 Turbo, GPT-4o, and GPT-o1) were tasked with classifying trials as positive or negative when provided with (1) conclusions only; (2) methods and conclusions; (3) methods, results, and conclusions; or (4) title and full abstract. LLM performance was evaluated against human annotations. Afterward, trials incorrectly classified as positive when the model was provided only with the conclusions but correctly classified as negative when provided with the whole abstract were analyzed for patterns that may indicate the presence of spin. Model performance was assessed using accuracy, precision, recall, and *F*_1_-score calculated from confusion matrices.

**Results:**

Of the 250 trials, 146 (58.4%) were positive, and 104 (41.6%) were negative. The GPT-o1 model demonstrated the highest performance across all conditions, with *F*_1_-scores of 0.932 (conclusions only; 95% CI 0.90-0.96), 0.96 (methods and conclusions; 95% CI 0.93-0.98), 0.98 (methods, results, and conclusions; 95% CI 0.96-0.99), and 0.97 (title and abstract; 95% CI 0.95-0.99). Analysis of trials incorrectly classified as positive when the model was provided only with the conclusions revealed shared patterns, including absence of primary end point results, emphasis on subgroup improvements, or unclear distinction between primary and secondary end points. These patterns were almost never found in trials correctly classified as negative.

**Conclusions:**

LLMs can effectively detect potential spin in oncology RCT reporting by identifying discrepancies between how trials are presented in the conclusions vs the full abstracts. This approach could serve as a supplementary tool for improving transparency in scientific reporting, although further development is needed to address more complex trial designs beyond those examined in this feasibility study.

## Introduction

Randomized controlled trials (RCTs) represent the gold standard for evaluating interventions in oncology [1]. However, the reporting and interpretation of trial results can be subject to inconsistency and “spin”—the presentation of results in a way that may mislead readers about the true efficacy of interventions [2]. This can, for example, be accomplished by emphasizing secondary end points or subgroup analyses when primary end points are not met. While most research that has looked at the topic has found a substantial prevalence of spin, the exact number varies as it is not always straightforward to differentiate between what constitutes a balanced and comprehensive presentation of the results and what may be an attempt to mislead the reader [3].

The presence of spin has important implications. Clinicians, policymakers, and even patients often rely heavily on abstracts and conclusions when interpreting trial findings, as full-text analyses are time-consuming and not always accessible. Therefore, misrepresentation of results might contribute to overly optimistic perceptions of treatment benefits, potentially influencing clinical decision-making, guideline development, and even the allocation of research funding. Given the increasing complexity of cancer care and the rapidly expanding volume of clinical trials, ensuring clarity and accuracy in scientific reporting is crucial to avoid bias in evidence synthesis and translation into practice.

The growing capabilities of large language models (LLMs) could constitute a standardized way to determine the presence of spin. If an abstract is clearly written, a state-of-the-art LLM should be able to determine whether its primary end point was met. As multiple studies have identified the conclusions as the most frequent source of spin [4,5], we hypothesized that trials which are correctly classified as negative—defined as trials that did not meet their primary end point—by an LLM when provided with the title and abstract, but incorrectly classified as positive when provided with only the conclusions, would be likely to contain some form of spin. Therefore, the aim of this study was to evaluate whether LLMs can reliably classify oncology RCTs as positive or negative and whether discrepancies between conclusion-only and full-abstract classifications can help identify patterns consistent with spin.

## Methods

### Overview

Randomized controlled oncology trials from 7 major medical journals (*British Medical Journal*, *Journal of the American Medical Association*, *Journal of the American Medical Association Oncology*, *Journal of Clinical Oncology*, *The Lancet*, *The Lancet Oncology*, and *The New England Journal of Medicine*) published between 2005 and 2023 were randomly sampled by downloading the available abstracts for the time frame via PubMed in a text file and parsing the abstracts using regular expressions. These 7 journals were selected because they publish a large and consistent volume of oncology RCTs and are widely regarded as leading general or oncology-specific medical journals. The 2005 to 2023 range was chosen to capture contemporary trial reporting practices while ensuring sufficient volume across all selected journals. To avoid edge cases for this feasibility study, it was decided to limit the eligible trials to designs with exactly 2 arms and 1 primary end point.

We aimed to sample 250 trials as this number ensured a sufficiently large dataset for the feasibility analysis while remaining feasible for manual dual annotation. Trials were sampled by creating a randomized list of all retrieved abstracts. Two authors (CK and PW) then started the annotation from the top of the random list and stopped after 250 two-arm, single–primary end point oncology trials had been annotated. No journal-level quotas were applied.

The purpose of the annotation was to establish the ground-truth classification—whether the trial met its primary end point—against which model predictions could be evaluated. The annotation was conducted in a 2-step process. After annotating the first 20 trials, all samples were discussed to recognize potential differences in the annotation criteria. The remaining trials were annotated separately, and discrepancies were discussed after all trials had been annotated. A third author (DRZ) would have been responsible for judging disagreements that persisted after discussion. However, this was not necessary. The annotation was performed using the Prodigy tool (version 1.13.1; Explosion), which only showed the extracted abstract as text without any additional information such as authors or institutions. Only in cases in which the abstract did not clearly state the primary end point and its results did we refer to the full publication or protocol. Three commercially available LLMs, namely, GPT-3.5 Turbo, GPT-4o, and GPT-o1 (OpenAI), were then tasked with classifying the trials as positive or negative. The 3 models were chosen to investigate whether the inherent capabilities of the models would impact their suitability for the classification task (eg, simpler models requiring more explicit language to correctly identify trials) and, thus, their performance when trying to leverage differences in classification accuracy to detect unclear writing and spin. The decision to use OpenAI models was based on the prevalent use of these models at the time as well as the convenience of application programming interface access and lack of privacy concerns regarding the study data. The respective model snapshots were gpt-3.5-turbo-0125, gpt-4o-2024-11-20, and o1-2024-12-17. The LLMs were called via the application programming interface, with the temperature parameter set to 1. We refrained from performing multiple classification runs as a previous study from the same research group had shown very consistent performance by LLMs for both classification and named-entity recognition tasks, as long as the temperature was kept at or below 1.50 [6].

Each model was evaluated in 4 different rounds. In round 1, the models were only provided with the conclusions of the abstract. In round 2, the models were provided with the methods and conclusions of the abstract. In round 3, the models were provided with the methods, results, and conclusions of the abstract. In round 4, the models were provided with the title and the full abstract.

The following system prompt (ie, the fixed instruction provided to the model to define its task) was used: “You will be provided with the {section} of a randomized controlled oncology trial. Your task will be to classify if the trial was positive, i.e. if it met its primary endpoint, or negative, i.e. if it did not meet its primary endpoint. Your response should be either the word POSITIVE (in all caps) or NEGATIVE (in all caps).”

The “{section}” part was replaced with either “conclusion,” “methods and conclusion,” “methods, results, and conclusion,” or “title and abstract.” The user prompt (ie, the specific input text) was the corresponding title, abstract, or sections of the abstract.

The prompts were designed to be as explicit as possible regarding the definition of a positive trial to minimize ambiguity and ensure consistent model behavior across conditions. However, we did not conduct a systematic comparison of different prompts.

### Statistical Analysis

Interannotator agreement was calculated as the percentage of agreement divided by the total number of annotated trials.

The results were evaluated against the ground truth (ie, the human-annotated classification of whether the trial met its primary end point) by creating confusion matrices and computing several performance metrics to obtain a holistic picture of model performance. These included accuracy (the proportion of correctly classified trials among all trials), precision (the proportion of predicted positive trials that were truly positive; equivalent to positive predictive value), recall (the proportion of truly positive trials that were correctly predicted as positive; equivalent to sensitivity), and *F*_1_-score (the harmonic mean of precision and recall). For completeness, specificity (true negative rate), and negative predictive value can also be derived from the confusion matrix but were not separately reported. The 95% CIs were estimated using normal approximation intervals. For the best-performing model, we further analyzed and categorized the trials that were incorrectly predicted as positive when provided with the conclusions but were correctly predicted as negative when provided with the title and abstract. For these trials, a single author (PW) reviewed the full conclusions and abstracts to categorize the patterns leading to incorrect classification (eg, omission of primary end point, emphasis on subgroup findings, or unclear distinction between end points). To contextualize these findings, we additionally selected 10 randomly chosen trials correctly classified as negative by GPT-o1 and performed the same qualitative assessment. All programming was performed in Python (Python Software Foundation; version 3.13.2) using, among others, the *pandas* (version 2.2.3) and *openai* (version 1.67.0) packages.

### Ethical Considerations

This study used publicly available abstracts from published clinical trials. All data were deidentified and contained no patient-level information; therefore, ethics approval was not required.

## Results

Interannotator agreement was 97.2% (243/250). All of the disagreements were caused by simple mistakes and could be easily resolved during the discussion. Ultimately, 58.4% (146/250) of the trials were annotated as positive, and 41.6% (104/250) were annotated as negative.

**Figure 1.**
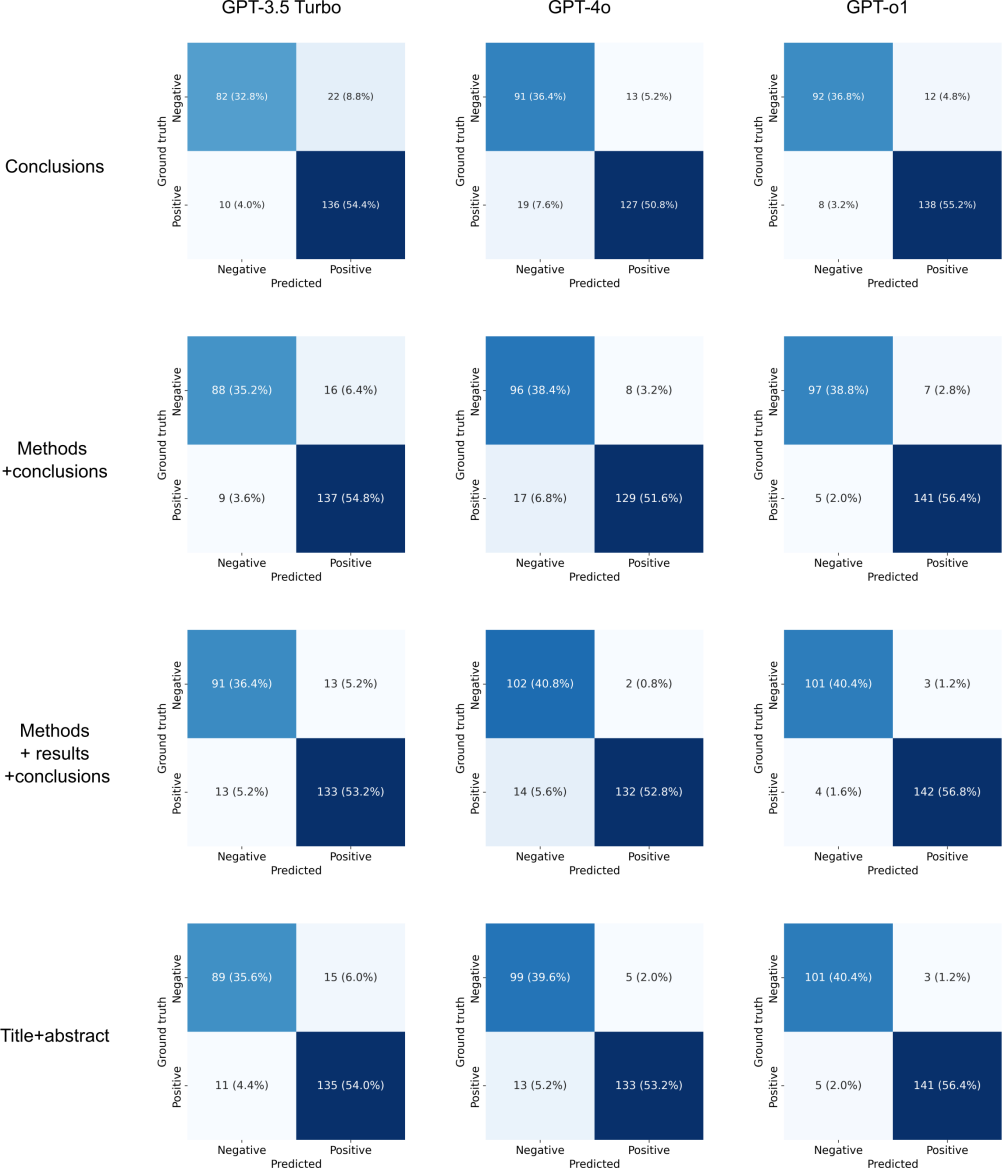
Confusion matrices. Classification performance of GPT-3.5 Turbo, GPT-4o, and GPT-o1 when predicting whether a trial was positive or negative based on different sections of the abstract.

The performances of the models when provided with different sections of the abstract are shown in Figure 1 and Table 1. GPT-o1 exhibited the best performance in each round, with *F*_1_-scores of 0.932 (conclusions only), 0.96 (methods and conclusions), 0.98 (methods, results, and conclusions), and 0.97 (title and abstract). GPT-4o’s *F*_1_-scores across the 4 rounds were 0.89, 0.91, 0.94, and 0.94, respectively. GPT-3.5 Turbo exhibited *F*_1_-scores of 0.89, 0.92, 0.91, and 0.91, respectively.

**Table 1.** Classification performance. Accuracy, precision, recall, and *F*_1_-score for GPT-3.5 Turbo, GPT-4o, and GPT-o1 when predicting whether a trial was positive based on different sections of the abstract.

	Accuracy (95% CI)	Precision (95% CI)	Recall (95% CI)	*F*_1_-score (95% CI)
Conclusions only				
GPT-3.5 Turbo	0.87 (0.83‐0.91)	0.86 (0.82‐0.90)	0.93 (0.90‐0.96)	0.89 (0.86‐0.93)
GPT-4o	0.87 (0.83‐0.91)	0.91 (0.87‐0.94)	0.87 (0.83‐0.91)	0.89 (0.85‐0.93)
GPT-o1	0.92 (0.89‐0.95)	0.92 (0.89‐0.95)	0.95 (0.92‐0.97)	0.93 (0.90‐0.96)
Methods+conclusions				
GPT-3.5 Turbo	0.90 (0.86‐0.94)	0.90 (0.86‐0.93)	0.94 (0.91‐0.97)	0.92 (0.88‐0.95)
GPT-4o	0.90 (0.86‐0.94)	0.94 (0.91‐0.97)	0.88 (0.84‐0.92)	0.91 (0.88‐0.95)
GPT-o1	0.95 (0.93‐0.98)	0.95 (0.93‐0.98)	0.97 (0.94‐0.99)	0.96 (0.93‐0.98)
Methods+results+conclusions				
GPT-3.5 Turbo	0.90 (0.86‐0.93)	0.91 (0.88‐0.95)	0.91 (0.88‐0.95)	0.91 (0.88‐0.95)
GPT-4o	0.94 (0.91‐0.97)	0.99 (0.97‐1.00)	0.90 (0.87‐0.94)	0.94 (0.91‐0.97)
GPT-o1	0.97 (0.95‐0.99)	0.98 (0.96‐1.00)	0.97 (0.95‐0.99)	0.98 (0.96‐0.99)
Title+abstract				
GPT-3.5 Turbo	0.90 (0.86‐0.93)	0.90 (0.86‐0.94)	0.92 (0.89‐0.96)	0.91 (0.88‐0.95)
GPT-4o	0.93 (0.90‐0.96)	0.96 (0.94‐0.99)	0.91 (0.88‐0.95)	0.94 (0.91‐0.97)
GPT-o1	0.97 (0.95‐0.99)	0.98 (0.96‐1.00)	0.97 (0.94‐0.99)	0.97 (0.95‐0.99)

We further analyzed trials that were incorrectly predicted as positive by GPT-o1 when the model was only provided with the conclusions but predicted correctly when provided with the title and abstract. Of these 10 trials, 6 (60%) did not mention the primary end point in the conclusions [7-12]. One mentioned an improvement in the primary end point in a subgroup [13]. One mentioned both improved secondary end points and the unimproved primary end point without specifying which was which [14]. The remaining 2 trials mentioned that one arm was superior to the other one without specifying that it was the control arm that showed improved results [15,16]. The list of trials is provided in Table 2.

**Table 2.** Trials that were incorrectly predicted as positive by GPT-o1 when the model was only provided with the conclusions but predicted correctly when provided with the title and abstract.

Title	Conclusions reported on primary end point	Possible reason for incorrect prediction
“Total Body Irradiation or Chemotherapy Conditioning in Childhood ALL: A Multinational, Randomized, Noninferiority Phase III Study” [15]	Yes	Conclusions mentioned that TBI^a^ plus etoposide showed improved overall survival. Therefore, the model likely thought that TBI plus etoposide was the intervention that was tested, whereas it was actually the control.
“Volasertib Versus Chemotherapy in Platinum-Resistant or -Refractory Ovarian Cancer: A Randomized Phase II Groupe des Investigateurs Nationaux pour l’Etude des Cancers de l’Ovaire Study” [7]	No	Primary end point was not discussed in the conclusions.
“High-Dose Therapy and Autologous Blood Stem-Cell Transplantation Compared With Conventional Treatment in Myeloma Patients Aged 55 to 65 Years: Long-Term Results of a Randomized Control Trial From the Group Myelome-Autogreffe” [14]	Yes	Conclusions mentioned both improved secondary end points and the unimproved primary end point without specifying which was which.
“Results of a Randomized Trial of Chlorambucil Versus Fludarabine for Patients With Untreated Waldenström Macroglobulinemia, Marginal Zone Lymphoma, or Lymphoplasmacytic Lymphoma” [8]	No	Primary end point was not mentioned in the conclusions.
“Bortezomib-Dexamethasone, Rituximab, and Cyclophosphamide as First-Line Treatment for Waldenström’s Macroglobulinemia: A Prospectively Randomized Trial of the European Consortium for Waldenström’s Macroglobulinemia” [9]	No	Primary end point was not mentioned in the conclusions.
“Adjuvant tamoxifen and exemestane in early breast cancer (TEAM): a randomised phase 3 trial” [12]	No	Primary end point was not mentioned in the conclusions.
“Addition of Bevacizumab to Bolus Fluorouracil and Leucovorin in First-Line Metastatic Colorectal Cancer: Results of a Randomized Phase II Trial” [10]	No	Primary end point was not mentioned in the conclusions.
“Oral ibandronic acid versus intravenous zoledronic acid in treatment of bone metastases from breast cancer: a randomised, open label, non-inferiority phase 3 trial” [16]	Yes	Conclusions mentioned the superiority of zoledronic acid. Therefore, the model likely thought that zoledronic acid was the intervention, whereas it was the comparator in this noninferiority study.
“Bcl-2 Antisense (oblimersen sodium) Plus Dacarbazine in Patients With Advanced Melanoma: The Oblimersen Melanoma Study Group” [13]	Yes	Improvement in the primary end point in a subgroup was mentioned.
“Efficacy and Safety of Trabectedin or Dacarbazine for Metastatic Liposarcoma or Leiomyosarcoma After Failure of Conventional Chemotherapy: Results of a Phase III Randomized Multicenter Clinical Trial” [11]	No	Primary end point was not mentioned in the conclusions.

a TBI: total body irradiation.

To confirm that those writing patterns were not equally frequent in trials correctly classified as negative, we also analyzed 10 random trials predicted correctly as negative by GPT-o1 and have provided the analysis in Multimedia Appendix 1. Of these trials, only 10% (1/10) did not mention the primary end point for the whole trial population in its conclusions but, instead, reported the results of the primary end point in a positive subgroup [17]. In total, 70% (7/10) of the trials explicitly mentioned that the primary end point failed to meet statistical significance or that the trial as a whole was negative or only mentioned the negative primary end point in their conclusions [18-24]. A total of 20% (2/10) of the trials mentioned both the primary end point and secondary end points or subgroups [25,26].

## Discussion

### Principal Findings

In this study, we evaluated the ability of 3 commercial LLMs to classify oncology RCTs as positive or negative based on different sections of trial abstracts. Our findings demonstrate that modern LLMs, particularly more advanced models, can achieve high classification accuracy even when provided with limited information. Our findings also support the hypothesis that trials that are correctly classified as negative by an LLM when provided with the title and abstract but incorrectly classified as positive when provided with only the conclusions are likely to contain patterns that may be interpreted as spin. While there is no ground truth of what constitutes spin, not mentioning the results for the primary end point at all in the conclusions, mentioning an improvement in the primary end point that only occurred in a subgroup, or mixed reporting of primary and secondary end points without clear distinction would be at least considered questionable by many readers [27]. Our findings also highlight that the LLM-based approach is not perfectly specific. In total, 20% (2/10) of the studies for which o1 was misled to believe they were positive when provided only with the conclusions had conclusions that clearly mentioned which arm had better outcomes. However, the LLM did not know which arm was the intervention and which arm was the control, so it assumed that the superior arm was the intervention arm. While this way of phrasing a conclusion may not be optimal for readability, it is certainly not an attempt at misleading the reader, who will still know which treatment yielded better results.

Therefore, our approach is likely not suitable as a fully automated solution. However, it demonstrated its potential to inform editors, reviewers, and authors alike of potential spin or unclear writing. The question of “Are the results for the primary endpoint clearly recognizable in the conclusion?” might serve as an alternative litmus test. Even though reviewers and journal editors are generally capable of recognizing questionable conclusions, we do believe that automated tools have value considering the ever-increasing list of items that have to be considered when conducting a careful review as they may, if implemented carefully, point toward parts of the manuscript that need increased attention. Another group of people who might benefit from a higher degree of automation are physicians who do not routinely read RCTs or have to do it in a situation in which they do not have time to fully digest all aspects of the research, such as in between patient consultations.

### Comparison to Prior Work

While research on LLMs and spin is still in its infancy, Yun et al [28] evaluated 22 LLMs and found that they are actually more susceptible to spin than humans. As LLMs are being used increasingly for screening and synthesizing scientific literature, this highlights the importance of improved detection of spin, preferably at the prepublication stage. However, the approach demonstrated in this study could also be leveraged as part of a screening pipeline to detect spin when trying to systematically analyze the literature in an automated fashion.

### Strengths and Limitations

This study has several strengths. The human annotation process was systematic, with independent dual review and consensus resolution, resulting in a reliable ground-truth dataset. Evaluating 3 LLMs of differing capability provided insights into how model complexity affects performance and sensitivity to unclear reporting. In addition, the structured comparison across 4 abstract conditions enabled us to isolate how specific sections of reporting contribute to misclassification.

This study has several limitations. First, the analysis was restricted to RCTs with 2 arms and a single primary end point. This constraint reduced complexity and helped ensure consistent interpretation but limits the applicability of our findings to trials with more complex designs, such as those involving multiple or co–primary end points. As noted in this paper, such designs introduce additional analytic considerations, for example, prespecified alpha splitting, that would have increased methodological heterogeneity and potentially confounded the evaluation [29]. Therefore, the restriction was deliberate, but it reduced generalizability.

Second, we did not include trials using analytical frameworks other than standard hypothesis testing, such as Bayesian designs [30]. Because these studies report results differently and may emphasize posterior probabilities rather than traditional statistical significance, the performance of LLMs in such contexts remains unknown. This limitation reflects the scope of the feasibility study rather than an inherent barrier of the method.

Third, it is uncertain whether the models had previously encountered some of the included abstracts during training. If so, prior exposure could have artificially increased performance, particularly when models were presented with only part of an abstract. Although this possibility cannot be fully eliminated for proprietary language models, our key analyses focused on discrepancies between conclusion-only and full-abstract predictions. These discrepancies are less susceptible to prior knowledge because recognizing internal inconsistencies requires examining the relationship between sections rather than retrieving memorized text. Nonetheless, this limitation may have influenced overall performance metrics.

Fourth, this study used a single, clearly defined prompt that specified what should be considered a positive or negative trial. While this approach ensured consistent instructions across models and conditions, it remains possible that different prompting strategies would yield different results. The choice of a single explicit prompt was intended to minimize variability, but it may limit insight into how models behave under alternative or less directive task formulations.

### Future Directions

Future work could extend this approach to more complex trial designs, including studies with multiple or co–primary end points, adaptive designs, or Bayesian frameworks, to determine whether LLM-based assessments remain reliable under conditions in which end point interpretation is less straightforward. Evaluating models from different vendors and open-source architectures may also help clarify how generalizable these findings are beyond the commercial systems examined in this study. In addition, refining prompting strategies or incorporating structured domain knowledge could improve model understanding of trial context, particularly in situations in which the distinction between intervention and control is not explicitly stated. Prospective integration of LLM-based screening tools into editorial workflows may help assess their practical utility in real-time manuscript evaluation. Finally, future studies may investigate whether LLMs can assist in promoting clearer reporting practices by providing automated feedback to authors during manuscript preparation.

### Conclusions

In conclusion, this study demonstrates that LLMs can highlight potential spin in oncology trial reporting by identifying inconsistencies between conclusions and full abstracts. These findings suggest a possible role for LLMs as supportive tools that draw attention to areas in which reporting may be unclear or incomplete. While not a substitute for expert review, such tools may help promote clearer communication of trial results. Further evaluation in more complex trial settings will be needed to determine how broadly this approach can be applied.

## Supplementary material

10.2196/78221 Multimedia Appendix 1 Analysis of 10 random trials predicted correctly as negative by GPT-o1.

## Abbreviations

LLM: large language model
RCT: randomized controlled trial
