# Supplementary material for: Large Language Models for Supporting Clear Writing and Detecting Spin in Randomized Controlled Trials in Oncology: Comparative Analysis of GPT Models and Prompts
Source: JMIR Cancer. 2026 Jan 21;12:e78221. doi: 10.2196/78221 (PMC12823016; doi:10.2196/78221)
Supplement: Multimedia Appendix 1 [file cancer-v12-e78221-s001.pdf]

Title	doi	Conclusion reports on primary endpoint	Comment
NGR-hTNF in combination with best investigator choice in previously treated malignant pleural mesothelioma (NGR015): a randomised, double-blind, placebo-controlled phase 3 trial	10.1016/S1470-2045(18)30193-1	Yes	Explicitly mentions that primary endpoint was not met
Randomized Phase III Trial of Induction Chemotherapy With Docetaxel, Cisplatin, and Fluorouracil Followed by Surgery Versus Up-Front Surgery in Locally Advanced Resectable Oral Squamous Cell Carcinoma	10.1200/JCO.2012.43.8820	Yes	Only mentions primary endpoint and that it failed
Tecemotide (L-BLP25) versus placebo after chemoradiotherapy for stage III non-small-cell lung cancer (START): a randomised, double-blind, phase 3 trial	10.1016/S1470-2045(13)70510-2	Yes	Conclusion begins with primary endpoint but also mentions subgroup
Randomized Phase III Trial of Capecitabine Compared With Bevacizumab Plus Capecitabine in Patients With Previously Treated Metastatic Breast Cancer	10.1200/JCO.2005.05.098	Yes	Conclusion begins with secondary endpoint then reports on primary endpoint
Placebo-Controlled Phase III Trial of Immunologic Therapy with Sipuleucel-T (APC8015) in Patients with Metastatic, Asymptomatic Hormone Refractory Prostate Cancer	10.1200/JCO.2005.04.5252	Yes	Explicitly mentions that primary endpoint did not meet statistical significance
Effect of Weekly Paclitaxel With or Without Bevacizumab on Progression-Free Rate Among Patients With Relapsed Ovarian Sex Cord-Stromal Tumors	10.1001/jamaoncol.2020.4574	Yes	Does not explicitly refer to primary endpoint but reports that the intervention achieved no clinical benefit
Comparison of Radiation With or Without Concurrent Trastuzumab	10.1200/JCO.20.02824	Yes	Explicitly mentions that the trial was negative

for HER2-Positive Ductal Carcinoma In Situ Resected by Lumpectomy: A Phase III Clinical Trial			
Predictors of Hand-Foot Syndrome and Pyridoxine for Prevention of Capecitabine-Induced Hand-Foot Syndrome: A Randomized Clinical Trial	10.1001/jamaoncol.2017.1269	Yes	Only mentions primary endpoint and that it failed
Tadalafil for Prevention of Erectile Dysfunction After Radiotherapy for Prostate Cancer	10.1001/jama.2014.2626	Yes	Only mentions primary endpoint and that it failed
Randomized, Double-Blind, Phase II Study of Ruxolitinib or Placebo in Combination With Capecitabine in Patients With Metastatic Pancreatic Cancer for Whom Therapy With Gemcitabine Has Failed	10.1200/JCO.2015.61.4578	No	Only mentions primary endpoint in a (positive) subgroup
